# Biomarkers of nutrition and stress in pregnant women with a history of eating disorders in relation to head circumference and neurocognitive function of the offspring

**DOI:** 10.1186/s12884-015-0741-7

**Published:** 2015-11-27

**Authors:** Saloua Koubaa, Tore Hällström, Kerstin Brismar, Per M Hellström, Angelica Lindén Hirschberg

**Affiliations:** Department of Women’s and Children’s Health, Karolinska Institutet, Stockholm, Sweden; Department of Clinical Neuroscience, Division for Psychiatry, Huddinge, Karolinska Institutet, Stockholm, Sweden; Department of Neuroscience and Physiology, Division for Psychiatry and Neurochemistry, Sahlgrenska Academy at University of Gothenburg, Gothenburg, Sweden; Department of Molecular Medicine and Surgery, Karolinska Institutet, Stockholm, Sweden; Department of Medical Sciences, Uppsala University, Uppsala, Sweden; Department of Obstetrics and Gynecology, Karolinska University Hospital, SE-171 76 Stockholm, Sweden

**Keywords:** Anorexia nervosa, Bulimia nervosa, Pregnancy, Ferritin, Thyroxine, Head circumference, Neurocognitive development, Offspring

## Abstract

**Background:**

Eating disorders during pregnancy can affect fetal growth and the child’s early development, but the underlying mechanisms have not been elucidated. The aim of the present study was to investigate serum biomarkers of nutrition and stress in pregnant women with previous eating disorders compared to controls and in relation to head circumference and early neurocognitive development of the offspring.

**Methods:**

In a longitudinal cohort study, pregnant nulliparous non-smoking women with a history of anorexia nervosa (*n* = 20), bulimia nervosa (*n* = 17) and controls (*n* = 59) were followed during pregnancy and their children’s growth and neurocognitive development were followed up to five years of age. We investigated maternal serum biomarkers of nutrition and stress (ferritin, cortisol, thyroid-stimulating hormone, free thyroxine, insulin, insulin-like growth factor I (IGF-I) and IGF binding protein 1) in blood samples collected during early pregnancy and compared between groups (ANOVA, LSD post-hoc test). The results were related to previous data on head circumference at birth and neurocognitive development at five years of age of the offspring (Spearman rank correlation or Pearson correlation test).

**Results:**

Serum levels of ferritin in the women with previous anorexia nervosa, but not in those with a history of bulimia nervosa, were significantly lower than in the controls (*p* < 0.01), and correlated strongly to impaired memory function in their children (rs = −0.70, *p* < 0.001). Maternal serum levels of free thyroxine were similar between groups but correlated positively to reduced head circumference at birth of the children in the bulimia nervosa group (r = 0.48, *p* < 0.05), and with the same tendency in the anorexia nervosa group (r = 0.42, *p* = 0.07), but not in the controls (r = 0.006). There were no significant differences in cortisol or the other biomarkers between groups.

**Conclusions:**

Low maternal serum ferritin in women with previous anorexia nervosa may be of importance for impaired memory capacity in the offspring at five years of age. Our results also indicate that thyroxin levels in pregnant women with previous eating disorders are positively associated with fetal head growth.

**Electronic supplementary material:**

The online version of this article (doi:10.1186/s12884-015-0741-7) contains supplementary material, which is available to authorized users.

## Background

Eating disorders (ED) during pregnancy may negatively affect intrauterine growth of the offspring. Previous studies have shown that particularly anorexia nervosa (AN) is associated with higher rates of prematurity, small for gestational age (SGA) and lower birthweight [1–4]. Furthermore, in a longitudinal cohort study we previously reported reduced head circumference in infants of mothers with a history of AN or bulimia nervosa (BN) [1]. The reduced head circumference at birth was related to delayed neurocognitive development, particularly expressive language skills, at five years of age in the group of children of mothers with a history of ED [5]. We hypothesized that impaired nutrition and/or maternal stress during pregnancy may be the underlying mechanisms.

A number of maternal serum biomarkers have been associated with fetal growth and neurodevelopment. Iron is essential for normal fetal development of several vital organs including the central nervous system [6]. In support, iron deficiency has been related to preterm delivery, low birth weight of the offspring, as well as iron deficiency in the newborns [6]. We have previously shown an increased frequency of anemia in our cohort of pregnant women with a history of ED compared to controls [1].

Maternal stressors have been related to small head circumference and impaired cognitive performance in the offspring [7]. It has been reported that women with AN and BN have elevated circulating levels of neuroactive stress hormones like cortisol [8]. Furthermore, prenatal treatment with corticosteroids has been associated with neurodevelopmental abnormalities [9]. Hypothetically, maternal hypercortisolism may therefore affect brain development of the child.

There is also evidence that maternal thyroid hormone function is of great importance for neuropsychological development of the child. In a large study, Haddow and co-workers [10] were first to demonstrate that undiagnosed maternal hypothyroidism was related to impaired cognitive performance in the offspring at around eight years of age. Furthermore, insulin and insulin-like growth factors are important regulators of developmental and cognitive functions of the fetus [11]. Diabetes during pregnancy can result in neurodevelopmental and neurocognitive defects [12]. Even in children of well-controlled diabetic mothers, fine and gross motor function could be impaired and higher rate of inattention and/or hyperactivity have been demonstrated [12].

In the present study, we report results on serum biomarkers of nutrition and stress (ferritin, cortisol, thyroid-stimulating hormone (TSH), free thyroxine (T4), insulin, insulin-like growth factor I (IGF-I) and IGF binding protein 1 (IGFBP1)) during early pregnancy in our cohort of women with a previous history of AN or BN in comparison to controls and relate these data to head circumference at birth and neurocognitive function at five years of age of the child.

## Methods

### Participants

In this longitudinal cohort study, the initial study population consisted of 49 women with a history of ED (24 AN, 20 BN, 5 unspecified eating disorder) and mean age ± SD 29.3 ± 4.6 years, and 67 control women aged 30.0 ± 3.7 years [1]. All women were nulliparous, non-smokers and conceived spontaneously. They were recruited in early pregnancy (gestational week 10) from 13 prenatal clinics in the northwest area of Stockholm and followed throughout pregnancy and up to three months post-delivery [1]. The women with a history of ED were initially diagnosed by interview according to the DSM-IV diagnostic criteria [13] and the diagnosis was further confirmed from medical records when available. The mean duration of ED was nine years (range 3–15 years) and the duration of recovery before study recruitment was 3.2 ± 3.0 (mean ± SD) years. During the same time period, 68 controls were recruited from the same prenatal clinics by interview, using the same inclusion criteria as for the patients but without a history of ED. Power calculation for the initial study population was based on birth weight as the primary outcome variable and the sample size was calculated to detect a difference between patients and controls of approximately 10 % with 80 % power [1].

In all women, a routine blood sample was collected during early pregnancy (gestational week 10) in connection with the first visit at the prenatal clinic for screening of infections (hepatitis B, human immunodeficiency virus infection, syphilis, rubella) and blood typing. After analysis, remaining serum was stored at the biobank of Karolinska University Hospital. All women gave their consent for the sample to be used for research purposes. Biobank samples from 37 women with a history of ED (20 AN, 17 BN) and 59 controls were available for analysis of biomarkers of nutrition and stress.

Neurocognitive development of the children was investigated at the age of five years [5] using a validated parent questionnaire, The Five to Fifteen (FTF) [14–17], which was delivered by mail and completed by the mothers. The FTF has relatively high internal consistency and an accetable to excellent inter-rater and test-retest reliability [14]. The complete questionnaire consists of 181 items on neurocognitive development divided into eight domains (motor skills, executive functions, perception, memory, language, learning, social skills and emotional/behavioural problems) and their subdomains, but for children who are five years old the domain learning is not used. Higher scores reflect difficulties in neurocognitive function.

The present research protocol was approved by the local Committee of Medical Ethics at the Karolinska University Hospital (2011/815-32) and the Biobank of Karolinska University Hospital (BbK-00682). Written informed consent was obtained from all women.

### Analytical methods

Serum levels of ferritin were analyzed by a routine chemiluminescence assay at the Department of Clinical Chemistry, Karolinska University Hospital. The detection limit was 0.2 μg/L and the total coefficient of variation (CV) was 8.6 %. Serum cortisol was analyzed using the instrument Modular E170/Cobas E (Roche Diagnostics, Mannheim, Germany). The CV for total cortisol was 3.1 % at 284 nmol/L and 3.8 % at 750 nmol/L. The assay measures both the free and protein-bound cortisol. Serum levels of TSH and free T4 were measured with enzyme immunoassays involving direct chemiluminescence, as described previously [18]. Insulin was analyzed using a magnetic bead-based metabolic panel kit from Millipore, Billerica, MA, USA (Cat# HMHMAG-34 K-06). The plate reader was a Luminex MagPix (Millipore) and the plate washer was a Tecan Hydroflex (Tecan, Männedorf, Switzerland) fitted with a magnetic holder. The inter-and intra-assay coefficients of variation were 12.5 % and 8.3 %. Serum IGF-I was determined by radioimmunoassay following separation from IGF-binding proteins by acidic ethanol extraction and cryoprecipitation [19]. The concentrations of IGFBP1 in serum samples were determined by radioimmunoassay according to Póvoa and co-workers [20].

### Statistical analyses

All values are presented as means and standard deviations, medians and inter-quartile ranges (P25–P75) or as percentage. One-way analysis of variance (ANOVA) was used to compare the three groups AN, BN and controls followed by the LSD post-hoc test. Prior to these analyses, certain variables were log-transformed and reciprocal transformed to compensate for their positively skewed distributions. When comparing IGF-I levels between groups, differences in age were adjusted for utilizing an age-adjusted score for this variable (IGF-I SD-score). Associations between biomarkers of nutrition and stress in mothers versus head circumference and neurocognitive function in their children were calculated using Spearman rank correlation or Pearson correlation test. A two-sided *p*-value <0.05 was considered to be statistically significant.

## Results

Table 1 shows pregnancy and neonatal outcomes of the study population. The AN women had significantly lower maternal body mass index (BMI) at early pregnancy, lower maternal weight gain, increased frequency of anemia (Hb < 110 g/L) and conceived infants with smaller head circumference than controls. The BN women had increased frequency of hyperemesis and their infants had significantly reduced head circumference at birth. Nine women (24 %) had verified relapse of eating disorder during pregnancy, of which 8 were diagnosed with previous AN and 1 with past BN.

**Table 1:** Pregnancy and neonatal characteristics in pregnant women

	AN *n* = 20	BN *n* = 17	Controls *n* = 59
Age of the mothers, years	29.8 ± 5.6	29.4 ± 4.2	29.8 ± 3.9
Maternal BMI, kg/m^2^	18.9 ± 2.9 ^a^***^,^^b^**	21.9 ± 3.0	22.5 ± 2.8
Maternal weight gain, kg	10.0 ± 3.8 ^a^**^,^^b^**	12.9 ± 3.8	12.2 ± 2.7
Weeks of gestation	38.7 ± 2.1	39.0 ± 1.6	39.1 ± 1.8
Hyperemesis, %	20	47 ^a^**	10
Anemia, %	70 ^a^***^,^^b^*	29	12
Birth weight, kg	3.2 ± 0.6	3.3 ± 0.7	3.5 ± 0.5
Birth length, cm	49.5 ± 3.1	50.2 ± 2.1	50.2 ± 2.4
Head circumference, cm	33.6 ± 1.6 ^a^***	33.8 ± 0.88 ^a^**	35.2 ± 1.6
SGA, %	10	12	3

Groups of mothers with a history of anorexia nervosa (AN) or bulimia nervosa (BN) and controls. Values are mean ± SD or percentage

Significant differences between groups are indicated: * *p* < 0.05, ** *p* < 0.01, *** *p* < 0.001

^a^AN or BN versus Controls, ^b^AN versus BN

BMI, body mass index

SGA, small for gestational age

**Fig. 1:**
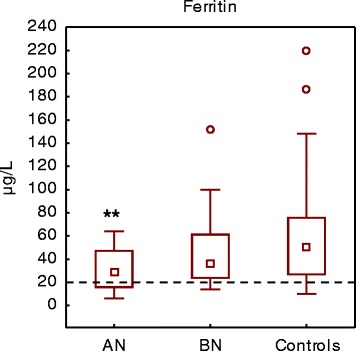
Serum levels of ferritin in the group of mothers with a history of anorexia nervosa (AN) (*n* = 20) or bulimia nervosa (BN) (*n* = 17) and controls (*n* = 59). The dashed line indicates the normal lower range for pregnant women. Values are medians and inter-quartile ranges (P25–P75). **p < 0.01 in comparison to controls

**Fig. 2:**
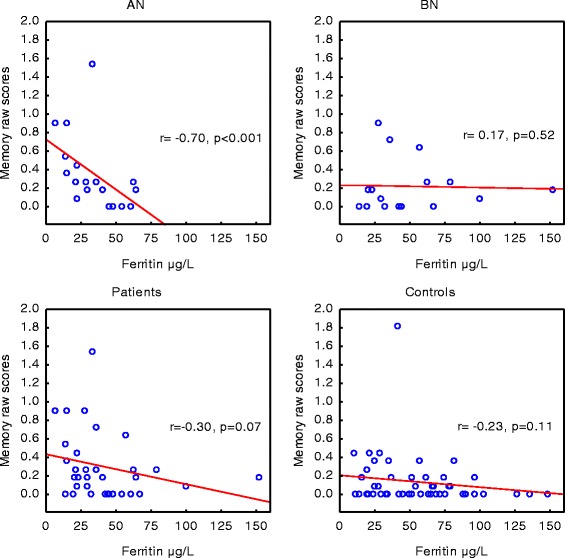
Correlations between maternal serum levels of ferritin and raw scores of memory function of the offspring at five years of age. Groups of mothers with a history of anorexia nervosa (AN) (*n* = 20), bulimia nervosa (BN) (*n* = 17), the combined groups of patients (*n* = 37) and controls (*n* = 59). Higher scores reflect impaired function

The combined group of AN and BN had lower ferritin levels than the controls (32 (21–54) vs 51 (26–76) μg/L, *p* < 0.05). This was also true for the AN group (*p* < 0.01) but not the BN group (Fig. 1). Twenty percent of the patients (5 AN and 3 BN) and 12 % of the control women had ferritin values below the normal range for pregnant women (<20 μg/L). In the combined groups of patients and controls, there was a positive correlation between serum ferritin and head circumference of the offspring at birth (r_s_ = 0.21, *p* < 0.05). There were no significant associations between these variables in any of the separate groups. Serum ferritin in the mothers with a history of AN, but not in those with a history of BN, was significantly associated with memory impairment of the offspring at five years of age (r_s_ = −0.70, *p* < 0.001) (Fig. 2).

**Fig. 3:**
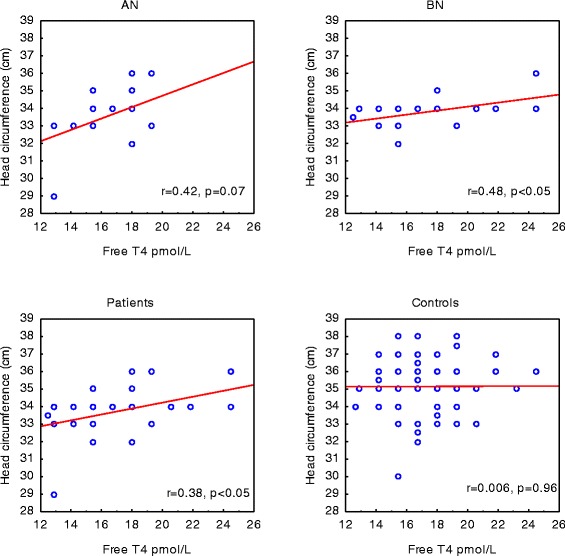
Correlations between maternal serum levels of free T4 and head circumference of the offspring at birth. Groups of mothers with a history of anorexia nervosa (AN) (*n* = 20), bulimia nervosa (BN) (*n* = 17), the combined groups of patients (*n* = 37) and controls (*n* = 59)

Cortisol levels were comparable between groups with the highest mean value in the control group (Table 2). In the AN group only, there was a positive correlation between cortisol and head circumference of the offspring at birth (r_s_ = 0.49, *p* < 0.05). Serum levels of TSH and free T4 were also similar between groups (Table 2). Ten percent of the women in the patient group and 8 % of the control women had TSH levels > 2 mE/L. Maternal TSH did not show any significant associations with measures of head circumference and neurocognitive function in any of the groups. However in the combined patient group, maternal free T4 correlated positively to head circumference at birth (r_p_ = 0.36, *p* < 0.05), as in the BN group (r_p_ = 0.48, *p* < 0.05), and with a similar tendency in the AN group (r_p_ = 0.42, *p* = 0.07), whereas there was no such correlation in the control group (r_p_ = 0.006) (Fig. 3).

**Table 2:** Serum levels of biomarkers of nutrition and stress in pregnant women

	AN	BN	Controls
	*n* = 20	*n* = 17	*n* = 59
Cortisol, nmol/L	401 ± 141	374 ± 121	418 ± 147
TSH, mIU/L	0.80 (0.51–1.08)	1.01 (0.62–1.17)	0.85 (0.51–1.30)
Free T4, pmol/L	16.7 ± 2.0	17.2 ± 3.8	17.4 ± 2.4
Insulin, pg/mL	100 (68–258)	94 (73–291)	138 (56–275)
IGF-I SD-score	−1.47 ± 0.80	−1.17 ± 0.95	−1.35 ± 1.02
IGFBP1, μg/L	149 ± 84	110 ± 55	120 ± 62

Groups of mothers with a history of anorexia nervosa (AN) or bulimia nervosa (BN) and controls. Values are mean ± SD or median and inter-quartile range

IGF-I, insulin like growth factor-I

IGFBP1, IGF binding protein 1

SD-score, age-adjusted score

T4, thyroxine

There were no significant differences in insulin, IGF-I SD-score and IGFBP1 levels between groups (Table 2). Maternal insulin correlated negatively to head circumference at birth in the BN group (r_p_ = −0.49, *p* < 0.05) but not in the AN group (r_p_ = −0.09, *p* = 0.70). Furthermore, serum levels of IGF-I SD-score correlated positively with head circumference of the offspring at birth in the patient group (r_s_ = 0.38, *p* < 0.05) but not in the separate groups.

## Discussion

In the present study, women with a history of AN were characterized by a low BMI in the 10^th^ week of gestation, a low weight gain during pregnancy, and a high prevalence of anemia in comparison to the BN and control groups. Nonetheless, birthweight and length of the offspring were not significantly different, but head circumference was reduced in the infant to mothers with a history of AN or BN. Our previous and other studies have shown that head circumference is positively associated with childhood cognitive performance [5, 21].

We found low levels of maternal serum ferritin in early pregnant women with a history of AN, which is in agreement with a high frequency of anemia in this group. Furthermore, there was a weak positive association between ferritin levels and head circumference of the offspring in the combined group of patients and controls. Other studies have found associations between both low and high levels of ferritin and various pregnancy complications, such as preterm birth, preeclampsia and intrauterine growth restriction [22, 23]. It is well established that serum ferritin during pregnancy is an indicator of maternal iron stores, with low levels diagnostic of iron deficiency. However, under certain clinical conditions high levels of ferritin, particularly during the third trimester, may be secondary to acute-phase reaction and rather indicate an acute or chronic infection [22]. This may explain the previously reported diverse associations between maternal ferritin and pregnancy complications.

We have demonstrated earlier that children born to mothers with a history of ED, and particularly to those with previous AN, had significantly impaired motor skills, memory capacity, language skills and social skills than control children at five years of age [5]. In the present study of the same cohort, we found that low serum ferritin in the mothers with a history of AN, but not in those with a history of BN, was associated with impaired memory of the offspring at five years of age. These observations suggest that deficient iron stores in the AN mothers may be of importance for neurocognitive development of the infant. Furthermore, there seem to be different mechanisms for the development of neurocognitive impairment in children of mothers with a history of AN and BN.

Our study did not demonstrate maternal hypercortisolism in the mothers with previous ED. Furthermore, we found somewhat unexpectedly, a positive correlation between maternal serum cortisol and head circumference at birth in the AN group. This finding gives no support for our hypothesis of a link between a high maternal serum cortisol level in mothers with ED and a reduced head circumference in the offspring [1, 5]. It is known that anemia and iron deficiency can induce maternal and fetal stress which stimulates the synthesis of corticotropin – releasing hormone (CRH) [24]. Elevated CRH is a major risk factor for preterm labor and also increases fetal cortisol production which may inhibit growth of the fetus. Maternal neuroticism was found to increase the risk for fetal weight restriction and head circumference restriction [25]. Furthermore, prenatal maternal objective stress was positively related to lower cognitive and language abilities at five years of age of the offspring [26]. However, maternal prenatal stress and/or maternal serum cortisol are in some studies positively related to fetal weight and development but data are sometimes conflicting [7, 27].

We found no differences in maternal serum levels of TSH and free T4 between groups. Low maternal levels of free T4 in early pregnancy are related to a delay of expressive language and nonverbal cognitive ability in early childhood of the offspring [28]. Furthermore, increased maternal serum TSH is associated with lower intelligence and motor scores of the child [10, 28]. Our finding of a positive association between serum free T4 in pregnant women with a history of BN or AN and head circumference at birth of the offspring could be in line with the above results.

In this study, we found a positive correlation between maternal IGF-I and head circumference of the child in the patient group. A positive relationship between maternal serum IGF-I and birth weight of the infant has previously been reported [29]. In agreement, our result may further support a role of IGF in fetal growth. Furthermore, good control of diabetes during pregnancy is essential to improve fetal growth [30]. Pregestational, as well as, gestational diabetes are associated with lower offspring cognition and educational attainment [12, 31]. The finding of a negative correlation between maternal serum insulin and head circumference at birth may be in agreement with this although our mothers were not diabetic. However, this association was significant only in the BN group and not in the mothers with a history of AN.

There are several limitations with the present study. Determination of serum biomarkers was performed only in one blood sample collected during early pregnancy and the results may therefore not be representative for the course of pregnancy. The blood sample was collected under non-fasting conditions and not standardized. The sample size was also limited. Furthermore, the FTF questionnaire was based on parent information of the mothers. Although the FTF test has demonstrated high inter-rater and test-retest reliability and validity, we cannot exclude reporter bias due to the possibility of worries and feelings of guilt in the mothers with a history of ED. Strength of the study is the longitudinal cohort design of area-based consecutive sampling of women with previous ED and controls and that only nulliparous, non-smoking women were included. We were also able to correlate maternal biomarkers with previous data on head circumference of the offspring at birth and neurocognitive function in the children at five years of age.

## Conclusions

Low maternal serum ferritin in the AN group, but not in the BN group, seems to be of importance for impaired memory capacity in the offspring at five years of age. Women with a history of ED, and particularly of AN, should be recognized as at-risk patients for anemia during pregnancy and be adequately treated to prevent potential negative outcomes of the offspring. Furthermore, the positive association between serum free T4 in pregnant mothers with previous ED and head circumference of the offspring suggests that maternal thyroid hormone function also is important for the child’s head growth and cognitive development.

## Additional file

Additional file 1:
**STROBE Statement—checklist of items that should be included in reports of observational studies.** (DOC 85 kb)

## Abbreviations

AN: Anorexia nervosa
BMI: Body mass index
BN: Bulimia nervosa
ED: Eating disorders
FTF: The five to fifteen questionnaire
IGF-I: Insulin-like growth factor I
IGFBP1: IGF binding protein 1
SD-score: Age-adjusted score
SGA: Small for gestational age
T4: Thyroxine
TSH: Thyroid-stimulating hormone

## References

[1] Koubaa S, Hällstrom T, Lindholm C, Hirschberg AL (2005). Pregnancy and neonatal outcomes in women with eating disorders. Obstet Gynecol.

[2] Ekeus C, Lindberg L, Lindblad F, Hjern A (2006). Birth outcomes and pregnancy complications in women with a history of anorexia nervosa. BJOG.

[3] Micali N, Treasure J (2009). Biological effects of a maternal ED on pregnancy and foetal development: a review. Eur Eat Disorders Rev.

[4] Wentz E, Gillberg IC, Anckarsater H, Gillberg C, Rastam M (2009). Reproduction and offspring status 18 years after teenage-onset anorexia nervosa-a controlled community-based study. Int J Eat Disord.

[5] Koubaa S, Hällstrom T, Hagenas L, Hirschberg AL (2013). Retarded head growth and neurocognitive development in infants of mothers with a history of eating disorders: longitudinal cohort study. BJOG.

[6] Radlowski EC, Johnson RW (2013). Perinatal iron deficiency and neurocognitive development. Front Hum Neurosci.

[7] Sandman CA, Davis EP, Buss C, Glynn LM (2012). Exposure to prenatal psychobiological stress exerts programming influences on the mother and her fetus. Neuroendocrinology.

[8] Monteleone P, Luisi M, Colurcio B, Casarosa E, Monteleone P, Ioime R (2001). Plasma levels of neuroactive steroids are increased in untreated women with anorexia nervosa or bulimia nervosa. Psychosom Med.

[9] Spinillo A, Viazzo F, Colleoni R, Chiara A, Maria Cerbo R, Fazzi E (2004). Two-year infant neurodevelopmental outcome after single or multiple antenatal courses of corticosteroids to prevent complications of prematurity. Am J Obstet Gynecol.

[10] Haddow JE, Palomaki GE, Allan WC, Williams JR, Knight GJ, Gagnon J (1999). Maternal thyroid deficiency during pregnancy and subsequent neuropsychological development of the child. N Engl J Med.

[11] Setia S, Sridhar MG (2009). Changes in GH/IGF-1 axis in intrauterine growth retardation: consequences of fetal programming?. Horm Metab Res.

[12] Ornoy A (2005). Growth and neurodevelopmental outcome of children born to mothers with pregestational and gestational diabetes. Pediatr Endocrinol Rev.

[13] American Psychiatric Association (APA) (1994). Diagnostic and statistical manual of mental disorders, fourth edition (DSM-IV).

[14] Kadesjö B, Janols LO, Korkman M, Mickelsson K, Strand G, Trillingsgaard A (2004). The FTF (Five to Fifteen): the development of a parent questionnaire for the assessment of ADHD and comorbid conditions. Eur Child Adolesc Psychiatry.

[15] Korkman M, Jaakola M, Ahlroth A, Personen A-E, Turunen M-M (2004). Screening of developmental disorders in five-year-olds using the FTF (Five to Fifteen) questionnaire. A validation study. Eur Child Adolesc Psychiatry.

[16] Trillingsgard A, Damm D, Sommer S, Jepsen JRM, Ostergaard O, Frydenberg M (2004). Developmental profiles on the basis of the FTF (Five to Fifteen) questionnaire. Eur Child Adolesc Psychiatry.

[17] Bohlin G, Janols L-O (2004). Behavioural problems and psychiatric symptoms in 5–13 year-old Swedish children – a comparison of parent ratings on the FTF (Five to Fifteen) with the ratings on CBCL (Child Behavior Checklist). Eur Child Adolesc Psychiatry.

[18] Naessén S, Carlström K, Glant R, Jacobsson H, Hirschberg AL (2006). Bone mineral density in bulimic women-influence of endocrine factors and previous anorexia. Eur J Endocrinol.

[19] Bang P, Eriksson U, Sara V, Wivall IL, Hall K (1991). Comparison of acid ethanol extraction and acid gel filtration prior to IGF-I and IGF-II radioimmunoassays: improvement of determinations in acid ethanol extracts by the use of truncated IGF-I as radioligand. Acta Endocrinol.

[20] Povoa G, Roovete A, Hall K (1984). Cross-reaction of serum somatomedin-binding protein in a radioimmunoassay developed for somatomedin-binding protein isolated from human amniotic fluid. Acta Endocrinol.

[21] Heinonen K, Raikkonen K, Pesonen AK, Kajantie E, Andersson S, Eriksson JG (2008). Prenatal and postnatal growth and cognitive abilities at 56 months of age: a longitudinal study of infants born at term. Pediatrics.

[22] Tamura T, Goldenberg RL, Johnston KE, Cliver SP, Hickey CA (1996). Serum ferritin: a predictor of early spontaneous preterm delivery. Obstet Gynecol.

[23] Hou J, Cliver SP, Tamura T, Johnston KE, Goldenberg R (2000). Maternal serum ferritin and fetal growth. Obstet Gynecol.

[24] Allen LH (2001). Biological mechanisms that might underlie iron’s effects on fetal growth and preterm birth. J Nutr.

[25] Chatzi L, Koutra K, Vassilaki M, Vardiampasis A, Georgiou V, Koutis A (2013). Maternal personality traits and risk of preterm birth and fetal growth restriction. Eur Psychiatry.

[26] Laplante DP, Brunet A, Schmitz N, Ciampi A, King S (2008). Project Ice Storm: prenatal maternal stress affects cognitive and linguistic functioning in 5 1/2-year-old children. J Am Acad Child Adolesc Psychiatry.

[27] Bolten M, Wurmser H, Buske-Kirschbaum A, Papousek M, Pirke KM, Hellhammer D (2011). Cortisol levels in pregnancy as a psychobiological predictor for birth weight. Arch Womens Ment Health.

[28] Li Y, Shan Z, Teng W, Yu X, Li Y, Fan C (2010). Abnormalities of maternal thyroid function during pregnancy affect neuropsychological development of their children at 25–30 months. Clin Endocrinol.

[29] Olausson H, Löf M, Brismar K, Lewitt M, Forsum E, Sohlström A (2008). Longitudinal study of the maternal insulin-like growth factor system before, during and after pregnancy in relation to fetal and infant weight. Horm Res.

[30] El-Masry SA, El-Ganzoury MM, El-Farrash RA, Anwar M, Abd Ellatife RZ (2013). Size at birth and insulin-like growth factor-I and its binding protein-1 among infants of diabetic mothers. J Matern Fetal Neonatal Med.

[31] Fraser A, Nelson SM, Macdonald-Wallis C, Lawlor DA (2012). Associations of existing diabetes, gestational diabetes, and glycosuria with offspring IQ and educational attainment: the Avon Longitudinal Study of Parents and Children. Exp Diabetes Res.

